# Boosting Sustainable Agriculture by Arbuscular Mycorrhiza under Stress Condition: Mechanism and Future Prospective

**DOI:** 10.1155/2022/5275449

**Published:** 2022-12-29

**Authors:** Surya Chauhan, Sonam Mahawar, Devendra Jain, Sudhir K. Udpadhay, Santosh Ranjan Mohanty, Abhijeet Singh, Elina Maharjan

**Affiliations:** ^1^Department of Molecular Biology and Biotechnology, Rajasthan College of Agriculture, Maharana Pratap University of Agriculture and Technology, Udaipur 313001, India; ^2^Department of Environmental Science, V.B.S. Purvanchal University, Jaunpur 222003, India; ^3^All India Network Project on Soil Biodiversity-Biofertilizers, ICAR-Indian Institute of Soil Science, 462038, Bhopal, M. P, India; ^4^Department of Biosciences, Manipal University Jaipur, Jaipur, India; ^5^Central Department of Microbiology, Tribhuvan University, Kirtipur, Kathmandu, Nepal

## Abstract

Global agriculture is frequently subjected to stresses from increased salt content, drought, heavy metals, and other factors, which limit plant growth and production, deteriorate soil health, and constitute a severe danger to global food security. Development of environmentally acceptable mitigation techniques against stresses and restrictions on the use of chemical fertilizers in agricultural fields is essential. Therefore, eco-friendly practises must be kept to prevent the detrimental impacts of stress on agricultural regions. The advanced metabolic machinery needed to handle this issue is not now existent in plants to deal against the stresses. Research has shown that the key role and mechanisms of arbuscular mycorrhiza fungi (AMF) to enhance plant nutrient uptake, immobilisation and translocation of heavy metals, and plant growth-promoting attributes may be suitable agents for plant growth under diversed stressed condition. The successful symbiosis and the functional relationship between the plant and AMF may build the protective regulatory mechansm against the key challenge in particular stress. AMF's compatibility with hyperaccumulator plants has also been supported by studies on gene regulation and theoretical arguments. In order to address this account, the present review included reducing the impacts of biotic and abiotic stress through AMF, the mechanisms of AMF to improve the host plant's capacity to endure stress, and the strategies employed by AM fungus to support plant survival in stressful conditions.

## 1. Introduction

Mycorrhiza is the type of fungus which forms mutualistic relationship with more than 80% plants on earth. Mycorrhiza was firstly described by Frank [1] and name derived from the Greek “*mukes*” (fungus) and “*rhiza*” (root). Principally, mycorrhiza establishes the association with plant roots. Mycorrhiza has four orders such as *Glomerales*, *Diversisporales*, *Archaeosporales*, and *Paraglomerales* and consists of ten families [2]. Mycorrhiza was futher divided into two broad groups, i.e., ectomycorrhiza and endomycorrhiza, and among them, the most common association is endomycorrhiza also called AMF. The AMF principally colonize the root cortex region of the plant and develops an extramatrical mycelium that laterally used for mineral nutrients acquisition (mainly phosphorus) from the soil [3]. The extramatrical hyphae are the unit of AMF used for nutrient transportation into the fungus. However, another branch-like structure formed by AMF within plant cortical region called “arbuscules” is the main site of nutrient exchange between AMF and the plant.

In exchange, plants give the AMF a source of carbon and a place to live, establishing a mutualistic symbiosis. The nature of the AMF can depend greatly on the soil, plants, and fungi conditions [4]. The relationship between AMF and plant is highly intricate and necessitates the exchange of chemical signals that result in mutual recognition and the growth of symbiotic structures. Strigolactones, which are obtained from plants, and Myc factor, which is derived from AMF, are the two main factors involved in this process and are essential to symbiosis. As soon as the plant exuded the signal molecule, the fungus identifies it and the hyphal development towards the plant root is stimulated, which later formed an appressorium on the surface.

**Figure 1:**
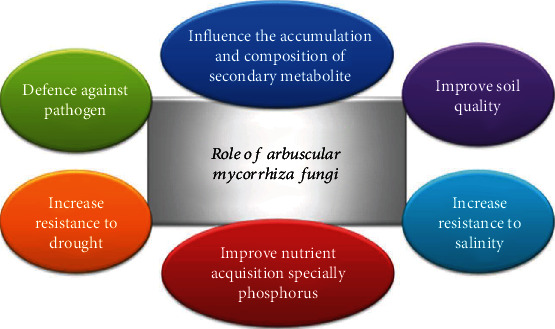
Role of arbuscular mycorrhiza fungi in stress mitigation.

After insertion of hyphae in root cortical region, AMF starts arbuscule formation, vesicle development (site of food storage), and hyphal growth colonizing the whole cortical region of 80% of the plant species except some of the vascular plant species belonging to plant families, viz., *Juncaceae*, *Caryophyllaceae*, *Chenopodiaceae*, *Cruciferae*, *Cyperaceae*, and *Chenopodiaceae*, which can not form mycorrhizae association. Genus *Glomus* (recently known *Funneliformis*) is the largest genera of AM fungi including important species, namely, *Funneliformis mosseae, F. etunicatum*, *F. viscosum*, *F. claroideum*, and *F. fasciculatum* [5]. Among these species, the oldest and most widely used *Rhizophagus irregularis* previously known as *Glomus intraradices* (*Glomeromycota*) is considered as the prime AMF and has been found in temperate and tropical regions of the world [6]. AMF symbiosis also proved as stress-mitigating agent and provides tolerant to plant against drought, salinity, and heavy metal, and the host plant's cability to combat biotic stresses is strengthened (Figure 1). Apart from said benefits, AMF also has some nonnutritive impact in improving soil texture [7].

## 2. Stress Biology (Abiotic and Biotic Factors)

Utilizing AMF may be a crucial strategy for promoting plant growth under stress and achieving sustainable agriculture goals. Examples of sustainable agricultural systems include using natural ways to increase output and food quality while reducing the use of artificial fertilizers, lowering input costs, and avoiding environmental harm. Previous studies state that the AMF successfully managed stress situations such as salinity, a lack of water, heat, heavy metals, and contaminated soils as proven by many studies in Table 1. This review covers abiotic stresses such as salinity, drought, temperature, and heavy metals. AMF is also a technique for reducing the detrimental effects of abiotic stress, such as drought, salinity, and biotic stress on plant growth [66].

### 2.1. Abiotic Stresses

**Figure 2:**
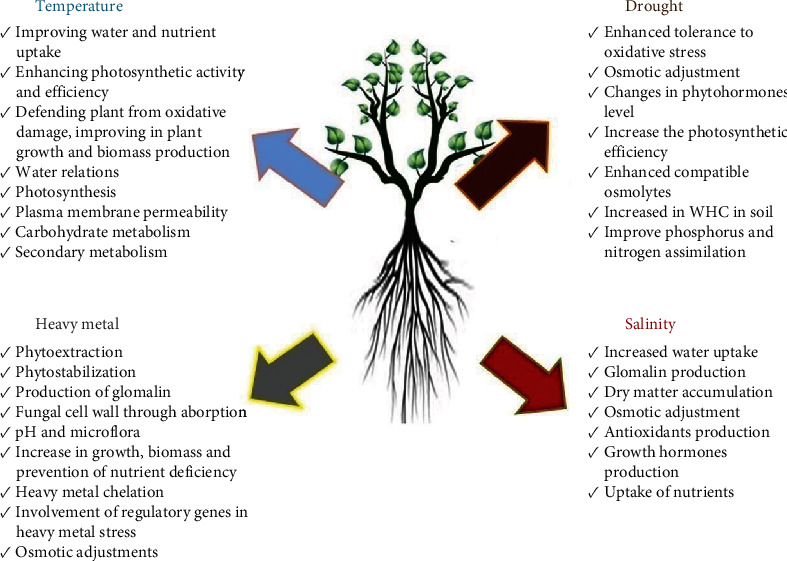
Schematic representation of the different mechanisms imparting abiotic stress tolerance in plants by arbuscular mycorrhiza fungi (AMF).

The abiotic stress, i.e., drought, salinity, heat, and cold, causes severe yield losses worldwide due to hampering severe distortion in the morphological, biochemical, physiological, and molecular parameters of plants [67–71]. AMF can play a significant role in the adverse abiotic stress to reduce the detrimental effects on plant growth that have been reported by numerous workers [72, 73]. Plants benefit from mycorrhizal symbiosis by reducing a variety of abiotic stresses as shown in Figure 2.

#### 2.1.1. Salinity and Its Effects on Plants

Salinity is one abiotic factor that is widely acknowledged to have a negative impact on agricultural productivity [74]. Around the world, salinity affects 20% of arable land and is slowly but steadily spreading, mostly in dry and semiarid regions. It was observed that in arid land, the precipitation is low and evaporation rate is high [66, 75, 76]. The major cause for salinity development is crop irrigation by saline water or high-salt-containing ground water and harsh climatic condition. Salinity stress induces undesirable changes in plant morphology and physiology which leads to retarded growth and development. Through multiple ways, plants get adversely affected by salinity stress which include the following:

- Deteriorating the vegetative growth and net absorption rate which results in lower production [77, 78]
- Stimulates the osmotic stress which affects the water status leading to decreased water use efficiency due to which water level reduces in plant, and ultimately, death of plant
- Augmented oxidative stress triggered by high concentration of Na^+^ and Cl^−^ which results into production of excessive reactive oxygen species (ROS) [79–82]
- Excess sodium ions (Na^+^) and chlorine ions (Cl^−^) cause ion poisoning which badly affects enzymes activity, functioning of cell membranes, and manufacture of plant hormones due to decrease in production of carbohydrates [83–86]
- Damages the photosynthetic efficiency of the plant by degrading the chloroplast function due to low uptake of magnesium and other nutrients [87, 88]
- The uptake of essential nutrients such as nitrogen, phosphorus, and potassium by the plant is hampered and imbalanced the plant nutrients [89]
- Other ways due to which salinity stress affects the plant development are poor germination, cuticle fragmentation leaf scorching or mottling, and shedding

#### 2.1.2. Role of AMF in Mitigating Salinity Stress

It is accepted worldwide that mycorrhiza fungi are proven to encourage host plant growth, yield and stress tolerance varying with AMF species, and host plants [90]. AMF possessed the capacity to develop and expand under high-salinity stress and provide the tolerance to host plant. The AM fungi withstand salinity stress by selecting several techniques that help the fungi and the host plant to grow under stress. AMF association with host plant is provided high-nutrient accumulation especially phosphorus, improve water uptake, activates plant hormones, and influences numerous physiological and biochemical aspects of plants in salinity conditions [90, 91]. Hashem et al. [10] investigated the effects of AMF species, viz., *Glomus etunicatum*, *Glomus intraradices*, and *Glomus mosseae* able to mitigate the salinity effects on growth and yield of *Cucumis sativus* L. Another report on AMF species *Claroideoglomus etunicatum* (formerly *Glomus etunicatum*), *Funneliformis mosseae*, and *Rhizophagus irregularis* had proven salinity mitigation ability in genotypes of wheat [92]. The effectiveness of AMF *Acaulospora laevis*, *Funneliformis geosporum*, *Funneliformis mosseae*, and *Cetraspora armeniaca* in wheat under salt stress was proven by Farghaly et al. [93]. Wheat-stressed plants with mycorrhizal inoculation had better growth characteristics and production. Comparatively to uninoculated plants, lipid peroxidation was dramatically reduced in mycorrhizal-inoculated plants. Alkalinity hindered catalase and peroxidase in wheat leaves and roots, but mycorrhiza increased the activity of these enzymes. To obtain desired AMF species for targeted stress mitigation, it would prefer to isolate from such type of stressed environments like drought, saline, and alkaline; hence, isolated AMF species can be more effective to mitigate the stress on host plant [94, 95].

The related mechanisms of alleviation by AMF under various stresses include.

*(1) Increased Uptake of Water by the Host Plant*. AMF alters plant physiology and changes morphogenetic characters of roots through its extensive hyphal network. Hyphae are used to absorb water and nutrients from the smallest soil pores, where root hair cannot reach or access due to improved root hydraulic conductivity at low-water potential [10, 88–98]. Ruiz-Lozano et al. [99] reported that the root hydraulic conductivity is effect by the synthesis of aquaporins, representing that there is a certain connection between the fungal activity and aquaporin synthesis under stress. Aquaporins are protein channels that regulate the flow of water and molecules like ammonium, CO_2_, urea, ions, amino acid, peptides, and glycerol inside the cells according to their gradient [100–102]. AMF also reduces meristem activity of root tips thereby leading to increased adventitious root formation and the host plant may be able to maintain water balance and nutrient uptake as a result of these AMF-mediated changes to the root architecture when exposed to salt stress. Studies revealed that AMF manage relatively higher water content in host plant as compared with non-AM plants [90, 103].

*(2) Glomalin*. Glomalin is a glycoprotein produced by AMF helped to soil particle aggregation and hence better the soil structure and properties [104]. Saline soil had poor in soil structure and loose soil aggregation in this perspective, Hammer and Rillig [72] reported that glomalin production by *Rhizophagus irregularis* was increased under salinity stress.

*(3) Dry Matter Accumulation*. Several studies have been reported earlier on photosynthetic activity and stomatal conductance was improved by the AMF inoculation under various stress [9, 73, 105, 106]. Another report revealed that AMF-associated sweet basil plants under salt stress had higher photosynthetic activity than nonstressed plants [107]. A higher quantity of sugars was detected in the leaves of mycorrhizal maize compared to nonmycorrhizal maize [108]. Another report on AMF-colonized plants under salt stress overextended levels of photosynthetic activity was higher than non-AMF-colonized plants, which shows mycorrhization is capable of completely balancing the stress [107].

*(4) Osmotic Adjustment*. AMF-colonized plant improves the water-retaining capacity of plants and positively regulating the osmotic balance and composition of carbohydrates. It was observed that AMF also improved the accumulation of many organic acids due to the osmoregulatory regulation of plant growth under salt stress conditions. AMF is good candidate for the accumulation of osmolytes like proline [109, 110], soluble sugar [111], betaine [112], and polyamines [90, 113] in host plants under various stresses. A study reported by Santander et al. [114] on AMF-colonized lettuce had high proline, nitrogen uptake, biomass production, and significant amount in ionic relations, chiefly reduced accumulation of Na^+^, when compared to no AMF-inoculated plant stress conditions.

*(5) Antioxidants Production*. Antioxidants are low-molecular weight molecule such as glutathione and ascorbate and they also have responsible for stress tolerance in plant under various stresses. AMF have the role in antioxidant activity improvement in stress condition. Mycorrhiza can regulate the level of reactive oxygen species (ROS) such as O^2-^, H_2_O_2_, OH^−^, and O^2^ by improving the activities of antioxidant enzymes such as superoxide dismutase (SOD), peroxidase (POD), ascorbate peroxidase (APX), and catalase enzyme (CAT) under stressed salt conditions [90, 114–116].

*(6) Production of Plant Growth Hormones*. Under stress condition, many signalling pathways are activated within the plants including mitogen-activated protein kinase, hormonal signalling, and fabrication of ROS [117–121]. Phytohormone signalling including cytokinins, auxin, ethylene, abscisic acid (ABA) [122], jasmonic acid (JA) [123], nitric oxide (NO) [124], salicylic acid (SA) [125], gibberellin (GA) [126], and strigolactone (SL) [127] are the signalling molecule which activated on AMF infection [125, 128]. AMF can also stimulate the changes in the contents of these growth hormones in plants infected by pathogens, therefore bringing defence response in plants.

(a)Augmented concentrations of cytokinins responsible for enhancing shoot growth [128, 129]. It was observed that plant hormones influence the phosphorus and other nutrient uptake through altering the root growth, plant physiology, morphology, and sugar signalling [130]. Against this AMF may change the expression of gene response for P-uptake in plants. Moreover, AMF promotes the p-responsive gene that affects the plant response to P-deficiency by the binding of transcription factors. The control of homeostasis, P-related pathways, and partitioning in plant cells is all impacted by such alterations [131]

(b)Strigolactones regulate the auxin transport in roots is affected by auxin carriers [132]. It revealed that the auxin might indirectly influence the symbiotic level as influenced by strigolactones. Henceforth, the role of auxin is correlated with AMF symbiosis [133]

(c)Arbuscule formation is regulated by the synthesis of ABA, which is necessary for AMF colonization in plant roots. In contrast, some AMF can accumulate considerable amounts of ABA [134], which is responsible for enhancing the root ABA concentrations of AM colonization plants [104]. Hameed et al. [134] has observed that AMF-mediated development in cytokinin amount results in a photosynthate translocation under salinity stress

(d)Strigolactones are playing a role in detection of AMF and development of symbiosis with host plant. Lopez-Raez [135] studied the strigolactones production in water-related stress-like drought and salinity with or without presence or absence of the AMF symbiosis. It is affirmed from the writing that in the absence of AMF colonization water deficiency upgraded the abscisic acid formation levels and down-directed strigolactone production. However, with AMF colonization, the strigolactone and ABA biosynthesis energise the host plants to elevate mutualism and support the host plant to performance under stress environment [135]

*(7) Uptake of Nutrients*. Under salinity, the nutrient uptake by AMF-colonized plant is also influenced because of the presence of high concentration of Na^+^ and Cl^−^ which showed chemically similar properties [136]. Potassium (K^+^) is a very important macronutrient to the plant growth and productivity having the similarity with Na^+^ ions. Therefore, in saline condition, Na^+^ uptake is high and K^+^ uptake decreases, hence high rate in ROS generation and level of photosynthesis decreased and finally plant growth is hampered. The harmfulness of Na^+^ is seen on account of its binding to the K^+^ sites at the plasma membrane, bringing about the failing of chloroplast, and influences enzyme activity and cell metabolism [137]. Apart from this, AMF colonization maintained the K^+^: Na^+^ ratio under high-salt stress condition [138–140]. AMF increases the uptake of several nutrients in host plant roots by disemination in dry soil when dispersion is low [141]. Plant obtains phosphorus in the form of Pi (inorganic orthophosphate) by the help of Pi transporters and this is major route of P-uptake in plants [142]. AMF-colonized plants have symbiotic route for acquisition of phosphate in P-deficiency environment [143]. If the phosphorus is rich in the soil environment, the AMF root colonization and development significantly reduced [144]. High Na^+^ and Cl^−^ ions are deposited in two specialized AMF organelles, i.e., vesicles and arbuscules which is developed for food and ion storage purposes. The arbuscules are tree-like in shape within the root cell and known for site of nutrient exchange with host plant, thus enhancing the plant capacity to absorb nutrients under stress [97]. Many researchers believe that fungi are unable to store ions in intraradical fungal hyphae or encode them in root vacuoles which are the reason for low level of both Na and Cl in plant tissues [145]. This specific ion take-up may reduce salt stress in host plant, partly through their nutritional enhancement [72]. Mycorrhizal plants further developed take-up of immobile nutrients such as Zn, P, and Cu [146–148]. AMF additionally adjust the plant physiology, upgrading plant development and tissue nutrients, allowing decreases in the utilization of synthetic fertilizers [66, 137, 149, 150]. Interactions of AMF with other soil microorganisms, such as plant growth-promoting bacteria (PGPR), also increase host plant survival, productivity, and yield, mitigating the effects of salinity [127, 151, 152]. Several researchers have reported [90, 153] that mycorrhizae increase salt tolerance through molecular and biochemicaland physiological changes in the properties of the host plant [94]. In experiments on grapevines, beans, and soybeans, mycorrhizal plants have shown remarkable accretion of proline, nonharmful, and defensive osmolyte, which assists the plant in maintaining osmotic equilibrium at low-water potential and act as an energy pool under salt stress [25, 93, 154].

#### 2.1.3. Drought and Its Effect on Plants

Drought is known as one of the most devastating stresses that remarkably diminishes crop yield worldwide and expected to enhancewith climatic changes [154]. Drought condition in which plant roots become inaccessible to water due to evaporation and intense heat remarkably negatively affect the plants survival [134, 155]. Drought has some bad impact on plant metabolic process like decrease in photosynthetic activity due to reduction in CO_2_ influx by stomatal closure, carbon partitioning [156], acceleration in generation of reactive oxygen species (ROS), oxidative stress [97, 157], reducing phosphate availability and other nutrient supply, and reduces crop quality. Global heating and lack of proper water-harvesting system worsened the drought conditions [39]. Therefore, it is demanding of sustainable strategies to make crop more resistant and to mitigate the adverse impacts of drought on crop yield.

#### 2.1.4. Role of AMF in Drought Stress Mitigating

- Extraradical mycelia of AMF enables extension of host root system to provide water along with mineral nutrients from the soil-root interface. Glomalin, polysaccharides, mucilages, and hydrophobins produced by AMF work to bind soil particles, resulting in soil aggregation with better water-holding capacity in soil [104]. Under drought stress, AMF increases root size and efficacy, leaf area index, and biomass [158, 159]
- Improve phosphorus and nitrogen assimilation in the colonized plants. Mycorrhizal P-transporters take up phosphate and convert it to polyphosphate, which is then transported in the direction of the plant. This pathway is made possible by the development of fungal aquaporins, which are crucial for osmoregulation [160]. Fungal hyphae in the host plant directly absorb NO_3_ or NH^4+^ to take up nitrogen. High levels of suitable osmolytes and protein synthesis are encouraged in host plants by increased nitrogen content [161]
- Increasing the photosynthetic efficiency of AMF inoculation, different studies revealed the role of AMF in protecting the photosynthetic activity by [162, 163] influencing the stomatal behaviour in the leaves of host plants, defining the water vapor efflux and CO_2_ gas exchange [164], leaf water potential (LWP) and relative water content (RWC), water use efficiency (WUE) of host plants, and thus enhancement of host water status in drought condition [32, 165–167]
- Changes in phytohormones level by AMF: During mycorrhization, abscisic acid (ABA) modulates the transpiration rate, root hydraulic conductivity, and aquaporin expression [168]. The higher level of abscisic acid concentration in host plant tissues induces stomatal closure for decrease of water loss and triggers many stresses responsive genes. Strigolactones (SLs) also play important role as phytohormones for host recognition signals for AM establishing in the host plant [135]
- Osmotic adjustment: AMF is a viable candidate for controlling the osmotic adjustment in the host plant because it promotes leaf sugar metabolism by altering the sugar metabolised enzymes, which significantly help colonized plants in osmotic adjustments. On exposure to a water-deficit environment, AMF also boosted the accumulation of suitable solutes such as sugars, proline, polyamines, glycine betaine, glutathione, and organic acids
- Increased resistance of plants to oxidative stress: Plants have evolved ROS scavengers in both their nonenzymatic (such as phenolic compounds, glutathione, ascorbic acid, alkaloids, carotenoids, and tocopherol) and enzymatic defence systems in response to stress [169]. As previously discussed, AMF has the ability to reduce oxidative damage in a variety of ways, including by increasing the production of phenolic compounds and secondary metabolites that detoxify ROS [170–172], increasing anthocyanin and carotene concentrations [167, 173], and ascorbic acid improvement of enzymatic antioxidants in mycorrhizal plants including superoxide dismutase (SOD), catalase (CAT), peroxidase (POD), guaiacol peroxidase, ascorbate peroxidise (APX), glutathione reductase (GR), and dehydroascorbate reductase (DHAR) [174]. Another strategy, AMF improves the plant defence from oxidative stress by lowering the amount of lipid peroxidation (MDA) and H_2_O_2_ accumulation [165, 166]. Many reports are available for the drought stress mitigation using AMF inoculation with several crops like wheat, barley, maize, soybean, strawberry, and onion [30, 166, 175]. Li et al. [106] have proved that AMF inoculation improved the growth and photosynthesis in *Leymus chinensis* and *Hemarthria altissima* plant by upregulation of antioxidant system. Under drought stress, Oliveira et al. [154] showed the symbiotic relationship between the soybean cultivar and AMF *Rhizophagus clarus*. These results suggest that AMF enabled the plant to lessen the physiological and growth impairments brought on by dry circumstances. Additionally, it was discovered that the crop under drought stress had increased levels of nitrogen and potassium as well as water potential and water use efficiency

*(1) Temperature (Thermal Stress)*. High temperature is also having negative impact of plant growth, physiology, and productivity [176]. Under low and high temperature, plant feels stress due to physiological and biochemical processes influenced. Temperature stress for plant is 0–15°C (low-temperature stress) and 45°C above (high-temperature stress) [177]. Many physiological and biochemical systems are disturbed when plants are exposed to low- or high-temperature stress. The various injuries caused by temperature stress include structural damage of cell membrane, peroxidation of membrane lipids, protein, toxic compounds production, plant vigor loss, seed germination inhibition, growth rate retardation, yield reduction, wilting, burning, abscission and senescence of leaves, damage to reproductive organs, and damage and discoloration of fruits. [77, 177–180]

#### 2.1.5. Role of AMF in Mitigating Temperature Stress

The probable mechanisms of AMF colonization are improve tolerance to the temperature stress of the host plants through many ways: (a) improving water and nutrient uptake, (b) enhancing photosynthetic activity and efficiency, (c) defending plant from oxidative damage, (d) improving in plant growth and biomass production, (e) water relations, (f) photosynthesis, (g) plasma membrane permeability, (h) carbohydrate metabolism, and (i) secondary metabolism.

*(1) Plant Growth and Biomass*. AMF increases the health of the colonized plants by improving its growth and biomass production. Many studies available on AMF plants grow better than non-AM plants under low- and/or high-temperature stress [181–184].

*(2) Water Relations*. AMF-infected plants showed better water status because of improved water withdrawal by the external hyphae [185] and greater hydraulic conductivity of the roots [185]. AMF is found to facilitate stomatal opening in leaves and water flow through the plants to the surfaces of the leaves that evaporate. [186]. Water uptake and hydraulic conductivity for the roots were made possible by the family of membrane intrinsic proteins known as aquaporins [187, 188]. Increased expressions of the PvPIP1;3 gene and PIP protein, as well as increased expression of the OsPIP1;1, OsPIP1;3, OsPIP2;1, and OsPIP2;5 genes, are all examples of how aquaporin gene expression is regulated in the host plants [189]. Plasma membrane intrinsic proteins (PIPs) also have the role in regulating the water transport by plant tissues [98]. AMF root colonization facilitated the plants to use water more efficiently [91, 177]. Studies on AM maize plants suggested improved plant water status at low- and high-temperature stress [176, 182, 189].

*(3) Photosynthesis*. Reports by Zhu et al. [182, 190] suggest that application of AM had higher CO_2_ assimilation capacity which implies higher photosynthetic rate (Pn) than the non-AM plants under temperature stress. Thus, in contrast to non-AM plants at suboptimal temperatures, AM colonization at the expense of extra carbon boosted plant development, hence inducing increased chlorophyll content [176, 182, 191].

*(4) Plasma Membrane Permeability*. Plasma membrane under temperature stress experiences increased electrolyte loss, as well as several compositional, structural, and other modifications that increase membrane permeability [178, 192]. Evelin et al. [90] reported that AM plants sustain higher electrolyte concentrations than non-AM plants through enhancing the membrane's stability and integrity.

*(5) MDH Peroxidation*. Peroxidation of malondialdehyde (MDH) present in plasma membrane occurs due to temperature stress [193]. Low-MDA content in AM plants maintains the fluidity of membrane and alleviates the peroxidation of membrane lipids [182, 183, 194, 195]. In cucumber (*Cucumis sativus*) plants, AM colonization at low temperatures promoted plasma membrane ATPase activities to control intracellular pH and to produce an electrochemical gradient for secondary active transport [196] and ATP buildup [184]. In addition to producing an electrochemical gradient for secondary active transport, ATPase can control intracellular pH [196].

*(6) ROS and Antioxidants*. Key reasons of injury to plants due to intense and cold temperature are oxidative stress. This oxidative stress brought by ROS (H_2_O_2,_ O_2,_ OH^−^) imbalances cell detoxification processes causing protein denaturation and enzyme inhibition which results in destruction of cell structure and function [197–199]. AM colonization under chilling stress lowers the extent of H_2_O_2_in cucumber leaves [184, 200]. One of the approaches by which AM fungi defend the host plants from temperature stress is by reducing H_2_O_2_ [201]. The AM symbiosis-induced low level of H_2_O_2_ may function as a signalling molecule in protective and adaptive responses [190]. Plants have developed a defence mechanism to forage ROS by antioxidant systems in order to reduce or avoid temperature stress-induced oxidative damage [194]. The antioxidant systems comprise of antioxidative enzymes like SOD and POD and compounds like tocopherols and polypohenols [90, 182, 199].

*(7) Osmotic Adjustment*. Osmotic adjustment is a crucial mechanism for tolerance in AMF plants growing in low- and high-temperature environments. Plants that are under temperature stress buildup compatible osmolytes like betaines, sugars, polyamines, acylated sterols, and proline that could reduce the osmotic potential in the cytosol and preserve the cells' positive turgor pressure [177, 178]. Numerous studies have demonstrated that AMF maize plants perform better than non-AM plants under low and high temperature [182, 195].

*(8) Carbohydrate Metabolism*. Sugars play important role in protecting AM plants to fight temperature stress and different types of sugars are involved to combat temperature stress depending on AM fungal species and host plant. First, they replace water molecules with lipid molecules to form hydrogen bonds, protecting and stabilising the plant cell membrane. Second, they serve as ROS scavengers. They assist in modulating plant stress responses, growth, and development that are linked to hormone signalling [177, 192, 202]. Thus, sugars act as cryoprotectant. Trehalose, the primary storage carbohydrate in AM fungus, resists damage from desiccation, which is a major factor in stress [90]. In response, fungal cells activate the trehalose metabolism enzymes transcriptionally and/or posttranscriptionally, which causes trehalose to build up [198, 203, 204].

*(9) Nutrient Uptake*. Under low- and high-temperature conditions, AM enhanced the host plant's nitrogen absorption. It is known that nutrient uptake is reciprocal since AM fungi absorb organic carbon from the plants and provide the hosts with nutrients [205]. The root epidermal cells and root hairs of plants mobilize and transport nutrients directly from the rhizosphere to the plants, while the extensive intraradical and extraradical mycorrhizal network serve as a channel for AM absorption [206]. According to numerous studies, AM fungi absorb, digest, and transmit large amounts of nitrogen through their extraradical mycelium in the host plant at either low or high temperatures [20, 190, 207–209]. According to Zhu et al. [190], mycorrhiza improves the maize plant growth in chilling stress.

*(10) Secondary Metabolites*. Plant secondary metabolites like strigolactones and flavonoids are crucial in spore germination and hyphal branching in AM fungi [210]. Plant secondary metabolites boost protein content and the plant immune system, helping it to withstand cold stress conditions [183]. Additionally, it has been demonstrated that AM fungi enhance secondary metabolites content in the host plants, including triterpenoids [90, 195]. Thus, the buildup of secondary metabolites and activation of enzymes relevant to secondary metabolism are connected to the antioxidant and defence systems of plants, implying that AM symbiosis increased plant tolerance to temperature stress. The beneficial effects of the *Glomus intraradices* symbiosis on watermelon *(Citrullus lanatus*) growth, physiological responses, and biochemical characteristics were investigated by Bidabadi and Mehralian [211]. AMF-inoculated watermelon seedlings outperform non-AMF-inoculated plants by increasing photosynthetic productivity and reducing oxidative damage brought on by chilling. Low-temperature stress on the roots of two different cultivars of barley (*H. vulgare*) was investigated by Hajiboland et al. [23]. In comparison to non-AMF-inoculated cultivars, AMF-inoculated barley cultivars show an increased phosphate uptake, plant growth, photosynthesis, and osmotic control at 5 and 25°C as well as improved postfreezing survival at −5°C.

#### 2.1.6. Heavy Metals and Its Effect

Increasing soil pollution has become major problem which leads to heavy metal accumulation in soil beyond a set limit due to human interference. Heavy metal deposition in soil is caused using agrochemicals, metal-containing wastes such sewage sludge and coal, and release of waste gases, water, and residue into the atmosphere. High levels of heavy metals (cadmium, palladium, mercury, etc.) are dangerous not only for the health of the soil and plants but also for human health since they can enter the food chain in a variety of ways and result in foods that are polluted with the heavy metals. Physical-chemical methods (such as soil rinsing and applying soil conditioner) and bioremediation, which uses bacteria and plants (phytoremediation), can both be used to clean up heavy metal-contaminated soil (microremediation and mycorrhizoremediation). Because they are more affordable, environmentally benign, and have fewer negative effects than chemical or physical cleanup, methods including phytoremediation, microremediation, and mycorrhizoremediation are recommended [212]. Heavy metal can impair the permeability and function of the plasma membrane, which affects the growth and development of plants. It can also cause oxidative stress, which negatively impacts cellular constituents and consequently plant tissues [213]. Due to the ageing of root tip cells in plants, heavy metals can also harm the roots causing them to appear yellow and wither.

*(1) Role of AMF in Mitigating Heavy Metal Stress*. AMF absorbed heavy metals through their hyphae either through chelating with other compounds in the cytoplasm or attach them to the cell wall and store them in the vacuole [213]. Additionally enhances the plant's defence mechanism to support growth and development, plant genotype, AMF type, and soil constituent type are all important factors in increasing plant tolerance to heavy metals [214]. AMF can lessen the stress of heavy metals on the growth of the host plant and promote effective plant growth. The AMF's strategies for reducing the negative environmental effects of heavy metals are the following.

*(2) Phytoextraction*. Phytoextraction uses the ability of plants to store and tolerate heavy metal in their plant parts and appealing for remediating contaminated soils [215]. The capacity of heavy metals to move through aerial plant organs and the formation of shoot biomass are two factors that affect the phytoextraction [216–218]. In *Zea mays* plants, AMF *Funneliformis mosseae* greatly boosts shoot weight and root length while decreasing the uptake of several metals like cadmium, cromium, nickel, and palladium [54].

*(3) Phytostabilization*. It boosts heavy metal plant uptake and distributes them to various plant sections [219, 220]. The different processes which explain the phytostabilization are as under:

- Numerous studies have demonstrated that AMF produces glomalin, a glycoprotein that is released into the soil and can immobilise a substantial amount of metal [221, 222]. According to research, one gram of glomalin can extract up to 4.3 mg of Cu, 0.08 mg of Cd, and 1.12 mg of Pb from contaminated sites. Glomalin is thought to reduce the risk of heavy metal toxicity to plants and other soil microbes. According to studies, Cd restores itself in the extraradical mycelium via binding to glomalin [223]
- The fungal cell wall is the first barrier to prevent heavy metal ions entering the plant [224]. According to reports, cortical cells and mantle hyphae have AMF-binding Cd and Zn in their cell walls [217, 218]. Its local concentration in the soil is decreased when heavy metals bind to chitin in the cell wall. Metal immobilization on extra- and intraradical fungal tissue has been demonstrated [219, 220]. According to reports, cortical cells and mantle hyphae have AMF-binding Cd and Zn in their cell walls [225, 226]
- pH and microflora: Studies reveal that the pH of rhizosphere soil treated with microbial agents is lower than that of aseptic agents. This difference in pH may be due to the activity of AM mycelium and AMF. AMF modifies the bacterial populations in the mycorrhizosphere and elevates soil pH. Due to the reduced availability of heavy metals to the host plant; mycorrhizosphere has lower concentrations of heavy metals in soil solution than that of nonmycorrhizal plants [227–229]. Significant contributions from plants are made through AMF, which modifies the bacterial populations in the mycorrhizosphere and elevates soil pH [227, 228, 230–232]
- Increase in growth, biomass, and prevention of nutrient deficiency: AMF accelerates physiological and morphological processes, enhancing the uptake of metal ions and decreasing metal toxicity in the host plants [233, 234]. Additionally, expand the plant's absorptive surface area outside the root hair zone to improve water use effectiveness and mineral uptake, both of which lead to increased biomass output [235]. This has been demonstrated in maize, soybean, and sunflower [225, 236, 237].
- Heavy metal chelation: AMF can be used to promote the regeneration and reconstruction of heavy metal-contaminated soil as well as help plants endure environmental stress [238]. The chelate of glycoprotein and metal enters the cell, reducing the content of removable metal elements in the soil [239].
- Involvement of regulatory genes in heavy metal stress: Four genes that are intended to be crucial in heavy metal stress resistance are (1) the GrosMT1 gene in *Gigaspora rosea*; (2) GinZnT1 *Glomus intraradices*' zinc transporter; (3) GmarMT1 gene in *Gigaspora margarita* and plants are shielded from oxidative stress brought on by metals by this gene which can encode MTs and regulate the oxidative reduction potential of fungi; and (4) GintABC1 can encode a polypeptide of 434 amino acids and actively taking part in the detoxification of copper and zinc [240, 241]. Metals primarily trigger or mediate the genes involved in fungal regulation, and these gene-mediated transport proteins are crucial for the uptake, transport, and tolerance of metals

*(4) Osmotic Adjustments*. Antioxidant enzymes eliminate free radicals and increase plant tolerance, just as was previously discussed in relation to the effect of osmotic adjustments in salinity and drought stress. In order to maintain the regular material and energy exchange of a plant's membrane, antioxidant enzymes like POD, SOD, and CAT could eliminate excess active oxygen in the plant. This could be used as a measure of the plant's stress resistance to adverse effects from heavy metals. The plant cell's antioxidant enzyme system lowers the level of membrane lipid peroxidation, inhibits ROS buildup, and promotes energy and material movement. In various crops, including wheat under aluminium stress [242], maize under silicon stress [243], rice [244], and alfalfa under cadmium stress [245], the AMF role in reducing heavy metal stress has been demonstrated.

### 2.2. Biotic Stress

Biotic stress includes resistance of AMF toward pests, insects, and pathogens such as pathogenic bacteria, fungi, virus, nematodes, and soil-borne pathogens. The advantages of mycorrhizal fungi in boosting plant tolerance to biological stresses caused by different pathogens interacting with varied plant species have been demonstrated among pathogenic fungus/oomycetes in numerous studies (such as*, Rhizoctonia* and *Fusarium*), nematodes (from the genera *Meloidogyne* and *Heterodera*), plant virus (such as TMV and PVY), phytopathogenic bacteria (*Xanthomonas campestris* etc.) [234]. To control plant infections and pests, biocontrol agents are based on biological microorganisms or derivative compounds and rely on the natural mechanisms that take place during the interaction between microorganisms and host plants [246]. In addition to establishing a stable and long-lasting attachment with the roots of many plant species, including legumes, rice, corn, soybeans, wheat, potatoes, tomatoes, and onions, AMF is well known for its application in biological control [205, 247]. Obligate biotroph AMF can have symbiosis with more than 80% of the plant species and offers several physiological implications to host plants one of which include development of resistance against pathogens and pest, thus also play an important role in management of soil-borne pathogens [248–250]. The effects of symbiosis vary among AM fungal isolates in terms of resistance or tolerance to biotic pressures for specific pathogen plant interactions. Reports have shown utilization of AM as a biocontrol agent in the integrated pest management (IPM) for disease control and focus on control of nematodes, bacteria, and fungi [153, 251–254].

#### 2.2.1. Mechanisms for Biocontrol of Biotic Stress Mediated by AMF

**Figure 3:**
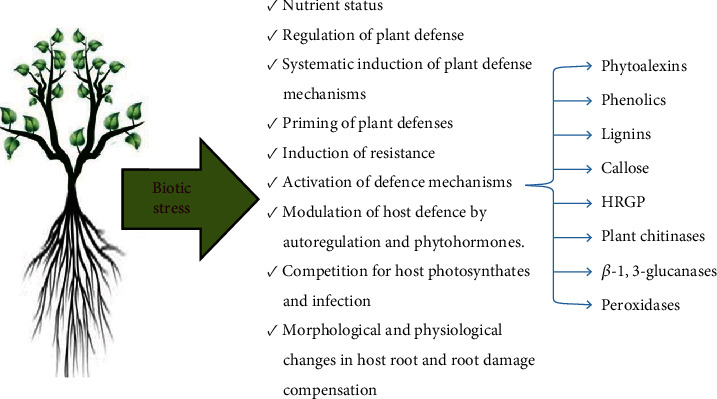
Schematic representation of the different mechanisms imparting biotic stress tolerance in plants by arbuscular mycorrhiza fungi (AMF).

Amalgamation of several modes is involved in the bioprotection via AM or mycorrhization rather than single [255].  Figure 3 depects the mechanism of AMF against biotic stresses. Symbiotic association between AM and plants has systemic effects in other parts of plants also, besides roots. In mycorrhizal plants' above-ground organs, two crucial systems that protect them against aerial diseases and pests may be involved. The vulnerability of the leaves to attackers may change as a result of changes in source-sink relationships and the uptake of extra nutrients by host plants [256]. The process of MIR (mycorrhiza-induced resistance) remains deceptive [257], despite the fact that regulation of plant defence mechanisms by AMF was thought to be one of the key processes engaged in the battle against aerial diseases and insects/pest [258]. The key mechanism is listed below.

#### 2.2.2. Nutrient Status of the Host Plant

Nutrient or mineral assimilation get enhanced by mycorrhizal symbiosis which leads to a robust plant due to which plant itself develops more tolerant or resistant to pathogen attack [259, 260]. Studies have shown that nutritional status improvement is not the sole mechanism for protection against pathogens. When plant diseases are absent, plant health increases, which is associated with the plant's capacity to continue producing in challenging circumstances. Regardless of their susceptibility to pathogenic illnesses, mycorrhizal plants are more resilient than nonmycorrhizal ones in terms of producing new weight thanks to the myorrhizal symbiosis. When compared to nonmycorrhizal plants, Dugassa et al. [261] found that *Oidium lini*-caused disease was less prevalent in mycorrhizal plants. These plants perform their functions more successfully than nonmycorrhizal cohorts because *R. irregularis* nutrition modification makes up for the pathogen damage and promotes plant growth.

#### 2.2.3. Regulation of Plant Defence Mechanisms During Mycorrhizal Establishment

The process of colonization is facilitated by the fungus' ability to pinpoint host roots by the exudation of strigolactones from the host roots, which are sesquiterpene signals that cause hyphal branching of AMF [256]. Because beneficial and dangerous fungi share certain molecular patterns known as microbe-associated molecular patterns (MAMPs), host plants initially mistake AMF for potential infections. [262]. MAMPs triggering plant defences in AMF are unknown [263], but their detection is what triggers a local temporary defence response of the plant immune system known as MTI for MAMP-triggered immunity. Activation of phenylpropanoid metabolism [264], transcript accumulation of hydrolytic enzymes like chitinases or glucanases [264], callose deposition [265], and increased salicylic acid (SA) synthesis in plant roots is some of the processes involved in MTI [266]. AMF produce effectors to control host signalling and locally decrease MTI, like how plant-pathogen interactions work, to enable a functional symbiosis with the host plant. According to Pel and Pieterse [267], plants are unable to distinguish between symbionts and diseases. Therefore, effector secretion is necessary to enable interactions between plants and advantageous microbes. Numerous anticipated fungal effectors may target diverse host-signalling mechanisms at different stages of arbuscular mycorrhizal symbiosis [268].

#### 2.2.4. AMF Induces Systematic Induction of Plant Defence Mechanisms

The presence of AMF can trigger defence mechanisms in plants' aerial parts, including the buildup of PR proteins, heat-shock proteins, and increased glutathione S-transferase and lipoxygenase activities [258, 269]. AMF colonization is expected to cause jasmonic acid (JA) buildup in plants. Cervantes-Gámez et al. [270] performed the first genome-wide investigation to find alterations in the expression of tomato plant genes in their leaves. AMF colonization is expected to cause jasmonic acid (JA) buildup in plants, one of the discovered hormonal changes. Regardless of the pathogenic state, Li et al. [260] showed that mycorrhizal soybean had a greater JA level than control plants. In mycorrhizal wheat plants, Mustafa et al. [271] found that in noninfected conditions, the genes for peroxidase, phenylalanine ammonia-lyase, and chitinase were upregulated.

#### 2.2.5. Activation of Plant Defences in Response to Pathogen or Pest Attack

The initial stages of the relationship between AMF and the host plant might prime the plant, resulting in a quick and potent systemic defence response to a subsequent pest or pathogen attack [250]. Split-root studies, which revealed immunity to non-AMF root components, validated the systemic action of plants [272]. Shoots were discovered to have increased expression of defence-related genes, including those that encode for enzymes like kinase and glycosyltransferases. When a pathogen infects a plant, MIR confers traits such as systemic resistance (SAR), and when beneficial bacteria colonize the roots, it confers traits such as induced systemic resistance (ISR) [250, 254, 273]. In fact, it is found that upon attack of pathogen, MIR is linked with SA-dependent genes in similarity with SAR-like priming, such as genes encoding a PR protein. This increase in the regulation of protective responses associated with SA pathways combines MIR with a response similar to SAR, unlike ISR, which is thought to occur without the aggregation of protective compounds antecedent to pathogen attack. Though like ISR, MIR, is generally regarded as a JA-based defence response. Furthermore, MIR provides protection against broad span of pathogens. These not only comprises of biotrophs which are prone to SA-mediated response but also nectrophs which are prone to JA or ethylene-mediated defences [273]. According to the findings, AMF plants are more vulnerable to biotrophs than necrotrophs, which results in the activation of JA-dependent defence mechanisms and the inhibition of SA-dependent defences in a mature mycorrhizal symbiosis, which is similar to the ISR response [249]. From an agricultural perspective, a response such as ISR to a mycorrhizal symbiosis may be more promising than SAR because in ISR, systemic plant protection initiates only upon pathogen attack but in SAR, even in its absence, systemic defence reponse works due to which the cost of survival is potentially more cost-effective in case of ISR than in SAR.

#### 2.2.6. Resistance Induction via Mycorrhizal Networks

Arbuscular mycorrhizal fungi (AMF) can broaden the induction of defence to nearby plants, in addition to their priming effects on a plant's aerial tissues. AMF can really spread from a specific mycorrhizal plant's root system to colonize the roots of other plants forming common mycorrhizal networks (CMNs) that link several plants of the same or different species [274]. In order to improve plant defence against pests and airborne pathogens, CMNs function as signalling pathways between plants. It was originally shown by Song et al. [275] that CMNs can serve as a defence route for communication between diseased and healthy tomatoes. Interplant signal transduction may occur through two different processes, the first of which is the continuous transfer of signalling molecules in fungal hyphae, and the second of which is the long-distance electrical signals that might form as a result of pathogen exposure and injury [276].

#### 2.2.7. Activation of Defence Mechanisms

Various essential defence systems that are either preformed or triggered in plants help to restrict the entry of dangerous pathogens. Induced defence mechanisms are a collection of plant processes that are activated in response to a pathogen attack in order to render the plant resistant. AM fungi can trigger specific defence genes of plant. Defence-related reactions in AM depends on stimulating a host response to a fungal attack which are suppressed in some way or kept at lower extent in accordance with the symbiotic interactions of especially activated plant and/or fungal mechanisms [277]. The different substances which are considered in association to AM formation are peroxidises, callose, phytoalexins, chitinases, phenolics, hydroxyproline rich glycoprotein (HRGP) lignins, callose, *β*-1,3-glucanases, and phytoalexins.

- Phytoa1exins are banefulcompound released at the infection site and compounds like coumestrol, isoflavonoids, and glyceollin were found in substantial quantity in mycorrhizal roots than in nonmycorrhizal roots [278, 279]
- Phenolics play an important part in protecting AM roots from fungal infections [280]
- Lignins: Lignification of vascular tissues and endodermal cell walls get stimulated by AM fungus [281, 282]
- Callose: Since it has been demonstrated to occur in the mycorrhizas of pea, tobacco, and leak but not maize, it is a variable feature of the cell wall response in all plants [276, 283, 284]
- Hydroxyproline rich glycoproteins (HRGPs): A large amounts of HRGP encoding mRNA were found to accumulate in mycorrhizal roots of celery [285]
- Plant chitinases: Even though there may be higher levels of endurance in combinations of some plant fungus, enhanced chitinase activity was seen during contacts between allium porrum, bean, and alfalfa roots with AM fungi in the early phases of root colonization. This activity was repressed to a level lower than in nonmycorrhizal roots throughout mycorrhizal establishment [264, 286–288]
- *β*-1,3-glucanases activity manifest a temporaryincrease in the early stages of root invasion. Although, reduced activity of *β*-1,3-glucanases is seen in the roots of the beans during the early colonial stages, but to a smaller extent in tomatoes. [287, 289]
- Peroxidases: In mycorrhizal roots, peroxidases are also more active and are associated with epidermal and hypodermal cells [290]

#### 2.2.8. AMF Symbiosis Modulates Host Defence Responses by Autoregulation and Phytohormones

Autoregulation is a phenomenon in which the plant can limit the colonization of AMF after the plant has become myorrhizal, which may affect its interaction with plant pathogens [255]. The levels of various phytohormones (like salicylic acid (SA), jasmonates (JAs), ethylene (ET), and abcisic acid (ABA) optimize defence responses in plants through a complex regulatory network. Their level appears to vary in mycorrhizal plants. Regarding pathogens that are biotrophic or necrotrophic, SA and JA play a significant role [291–293]. The SA and JA signalling pathways are coupled [119, 280, 282] most frequently antagonistically [294] and may explain enhanced resistance/susceptibility in AM plants [129, 294, 295].

#### 2.2.9. Plant Responses That Are JA-Dependent Are Primed by the AM Symbiosis

The basal defence system of plant in which preconditioning of plant tissues activates plant immune system upon attack is known as priming [296, 297]. Priming appears to be an approach followed by various advantageous microbes to increase plant resistance and thus evading a direct activation of defences which would be very costly to the host without provoking invaders [293, 298]. Priming related to systemic resistance induced by beneficial organisms is regulated by similar jasmonate signalling pathways [298]. The foundation of the primed state is JA accumulation, which may mediate the plant's “remembering” of prior difficulties [299]. Increased levels of jasmonates in mycorrhizal roots are evidence that they are important hormones in the AM symbiosis [129, 300].

#### 2.2.10. Infection and Competition for Host Photosynthates

For growth and survival, both mycorrhizal fungi and pathogens compete with one another for the host's photosynthetic products to reach the roots [301, 302]. High levels of C requirement can stop pathogen to grow by allowing access of photosynthates to mycorrhiza primarily [259]. AM fungi and pathogens develop in different cortical cells in the same root system which indicate competition for space. Despite having the same root system, the infections and the AM fungus often emerge in distinct cortical cells, suggesting some sort of spatial conflict [259]. By colonizing the roots, AM fungus protects the plant from pathogenic fungus and prevents it from spreading to the area of the roots where it has not yet done so [303].

#### 2.2.11. Morphological and Physiological Changes in Host Root and Root Damage Compensation

AM fungi develop a compact root system in the plants which comprises of short and adventitious roots leading to development of comparatively greater proportion of higher matured roots in the root system [304, 305]. AM fungi role in changing root framework do not induce resistance directly in plants but the factors associated with these alterations may be responsible [306]. There are different advantages which AM fungi provide to plant roots despite of pathogen [307] and nematode attack [307] which includes, enhancing continual functioning of roots for encouraging mycorrhizal fungus to absorb nutrients and water which leads to disease suppression and enhancing production of amino acid specially arginine which inhibits spore formation in pathogen [308].

#### 2.2.12. Mycorrhizosphere: A Biocontrol Zone

The term “mycorrhizosphere” refers to the area that mycorrhiza covers and explores. It is proposed that the mycorrhizosphere comprises of an environment beneficial to microbesantagonist to soil-borne pathogen development. Mycorrhizosphere can be considered as triple association of three components, viz., root-mycorrhiza-rhizobacteria and each component influence other components health, growth, and development. By releasing glycoproteins such as glomalin, mycorrhizal fungi encourage the growth of aggregates that offer stable microsites favourable to the development of roots and microorganisms [309]. It has been demonstrated that mycorrhizal fungi and plant growth-promoting rhizobacteria (PGPR), particularly phosphate-solubilizing and nitrogen-fixing bacteria, interact and work together in a synergistic manner. These PGPR boost the plant's access to phosphate and nitrogen, which fosters its growth and development and may help it better withstand the impact of pathogens on plant growth and production [310–314].

## 3. Effect of Biotic Stress on AMF

### 3.1. Plant Virus Diseases

According to Maffei et al. [315], the tomato plant's colonized AM fungus, *F. mosseae*, offers defence against the virus and has a protective impact against the tomato yellow leaf curl Sardinia virus (TYLCSV), which results in more pronounced mild symptoms and having less viral DNA.

### 3.2. Bacterial and Phytoplasma Diseases

AMF inhibits the growth of *Xanthomonas campestris* and protects against phytopathogenic bacteria, causing certain necrotic lesions in tomato plants colonized by *Rhizophagus irregularis* [270, 316] or in alfalfa infested by AMF [269].

### 3.3. Fungal Diseases

Different studies reveal the role of AMF in conferring resistance against fungi mainly from the genera *Colletotrichum* [265, 316, 317], *Alternaria* [258, 318, 319]*, Botrytis* [320–323], and *Fusarium* [261, 319]. It also confers resistance against oomycota and genus *Phytophthora* [260, 324–326]. The extent up to which AMF can provide protection against such pathogen largely depends upon the lifestyle of attackers, being the biotroph, necrotroph, or hemibiotroph [249, 250]. In the case of necrotrophic fungi, it appears that the presence of AMF makes plants more susceptible to diseases [318, 320]. The role of AMF was found contentious in case of hemibiotrophic fungi or oomycota since the lifestyle of hemibiotrophs is like biotrophic initially which switches to necrotrophic lately. Additionally, biotrophic phytopathogens have been reported to lack protection.

### 3.4. Pest Insects

Pest infestations appear to be impacted by AMF symbiosis and are dependent on the lifestyle and level of specializations of the insects. Generalist insects that appear to feed on a variety of hosts and are sensitive to plant defence responses are negatively impacted by the advent of AMF [327, 328]. The metabolism of volatile organic molecules and iridoid glycosides, which are involved in both direct and indirect plant protection, can be regulated by AMF [325]. Conversely, specialist insects act better on mycorrhizal plants, perhaps due to less susceptibility to plant defence responses, along with better nutritional value for mycorrhizal plants [329, 330].

### 3.5. Effects of AM Symbioses on Pathogens from the Soil

AM symbioses are crucial for reducing the harm that soil-borne pathogens can do. Numerous studies have showed a decrease in the frequency or severity of illnessesbrought on by a wide range of fungus, bacteria, and oomycetes, including *Verticillium* and *Rhizoctonia*, as well as oomycetes, such as *Phytophthora, Pythium*, and *Meloidogyne* [307, 330–332].

### 3.6. Effects of AM Symbioses on Root Parasitic Plants

Plants of the genera *Striga* and *Orobanche* are one of the most harmful agricultural pests of parasitic weeds which parasitize on various hosts around the world [333, 334]. Strigolactones are sprouting excitants for root parasitic plant's seed [335]. Decreased production of striglactones in a mutant of tomato associated with a reduced sensitivity for *Orobanche* [334]. Studies have shown that decrease in strigolactone formation can lead to reduction in occurrence of root parasitic plants on mycorrhizal plants.

## 4. Future Prospectives

The host plant's physiology is dramatically altered when AM symbiosis forms with plant roots, and this transformation has a long-lasting impact on the plant and its biotic interaction. Mycorrhizal symbioses essentially improve a plant's ability to withstand biotic stresses. Despite the fact that individual outcomes have always been reliant on the interaction between AMF and plant invaders, defence mechanisms against harmful organisms, which range from pathogenic bacteria to insect herbivores, have been amply demonstrated. According to research, this defence depends on both plant defence systems and nutritional enhancement or fundamental changes in the rhizosphere and roots. The route appears to be strongly dependent on jasmonate signalling; for example, mycorrhizal symbioses increase plants' defences against invaders. The principles of successful symbiosis and the functional relationship between the plant and the fungus are revealed, and this is highly intriguing. Finding protective regulatory components that coordinate the growth of mycorrhiza and mycorrhiza-induced resistance is the study's key challenge. This acknowledgement will pave the way for the creation of biotechnological blueprints to enhance the formation of mycorrhiza and the use of AMF in the integrated management of diseases and pests. More development is required to produce more mycorrhizal crops under diverse stress conditions. This may be appropriate for restoring land and soil in areas impacted by salinity or drought.

By producing glomalin, trehalose, proline, phytohormones, antioxidant enzymes, aquaporins, and higher nutritional content, colonization of AM fungus plays a valuable and effective role under abiotic stress conditions. The development of a sustainable agriculture system depends critically on the use of AMF in modern agriculture. All goals of sustainable agriculture, including a decrease in the use of synthetic fertilizers and other chemicals, will surely be aided by this. Utilizing AMF for agricultural applications will boost crop growth and production, surpassing the expanding consumption needs of the world population. Strong encouragement is required to ensure that eco-friendly technologies are widely used.

Future research in this field might concentrate on defining the crucial physiological and metabolic processes that occur in a variety of environmental contexts, as well as the host- and AMF-specific protein elements that control symbiotic relationship. Understanding how tolerance mechanisms are adjusted by AMF and the crosstalk is engaged to regulate plant efficacy would help to boost crop productivity. All of their interactions must be studied in order to comprehend how AMF works in nature as a bio-fertilizer for sustained agricultural output.

In the future, study the mechanisms underpinning the uptake, immobilisation, translocation, and metabolism of metals by plants. In addition, research on gene regulation and the theories supporting AMF's compatibility with hyperaccumulator plants should be done. It is necessary to conduct research on how to put mycorrhizal remediation of metal-contaminated soil into practise for it to be more beneficial and effective. More research is required to determine the best AMF with excluder, hyperaccumulator, and transgenic plant combinations. Excluder and hyperaccumulator plants can both thrive in environments with high concentrations of harmful metals. Excluder AMF and excluder plants might work well together to support the restoration of these contaminated sites. In order to address this, and based on research results from in vitro or pot tests, the role of AMF as plant growth promoters and excluders would be a tremendous aid for sustainable agriculture in stressed soils. It would also restore soil health and create new possibilities for future research.

## Figures and Tables

**Table 1:** Contribution of AMF in helping plants to cope with biotic and abiotic stress.

Host plant	AMF strains	Stress	Observed responses	References
*Solanum lycopersicum* L.	*Rhizophagus irregularis*	Salinity	Rise in growth hormones, newly formed root and shoot weight, leaf area, and leaf count	Khalloufi et al. [8]
*Leymus chinensis*	*Glomus mosseae*	Salinity	Enhancement in level of phosphorus, nitrogen, seedling weight, and water content	Jixiang et al. [9]
*Cucumis sativus* L.	*Claroideoglomus etunicatum*, *Rhizophagus intraradices*, and *Funneliformis mosseae*	Salinity	Increase in biomass, antioxidant enzymes, proline and phenol level, and mineral elements and decrease in uptake of Na ions	Hashem et al. [10]
*Medicago sativa*	*Glomus mosseae*	Salinity	Enhancement of biomass and nutrients uptake, accumulation of P over N and K, PSII and PSI system stomatal conductance, and ability to utilize CO	Shi-chu et al. [11]
*Glycine max* L. Merrill	*Claroideoglomus etunicatum*, *Rhizophagus intraradices*, and *Funneliformis mosseae*	Salinity	Improvement in plant and root system by increase in nutrient uptake. Reduction in lipid peroxidation and membrane damage by salt	Diagne et al. [12]
*Prunus dulcis* × *Prunus persica* hybrid	*Rhizophagus intraradices* and *Funneliformis mosseae*	Salinity	Enhancement of antioxidant enzymes, photosynthetic compounds, soluble sugars, and proline content	Shahvali et al. [13]
*Pisum sativum* L.	*Rhizoglomus intraradices*, *Funneliformis mosseae*, *Rhizoglomus fasciculatum*, and *Gigaspora* spp.	Salinity	Increase in biomass, chlorophyll content, nutrient uptake, accumulation of compatible osmolytes, and growth characters	Parihar et al. [14]
*Euonymus maackii* Rupr	*Rhizophagus intraradices*	Salinity	Enhancement in photosynthetic capacity, nutrient uptake, and antioxidant enzyme	Li et al. [15]
*Citrullus lanatus* L.	*Glomus mosseae* and *Gigaspora gigantean*	Salinity	Increase in leaf area, size and weight of fruits, root colonization, chlorophyll potassium, magnesium, iron zinc level, ROS, and ABA level osmotic potential antioxidant enzyme	Bijalwan et al. [16]
*Eucalyptus camaldulensis*	*Glomus* spp., *Gigaspora albida*, and *Gigaspora decipiens*	Salinity	Enhancement in photosynthetic pigment and reduction in leaf proline and decelerate the negative effects on physiological and biochemical parameters	Klinsukon et al. [17]
*Zea mays*	*Rhizophagus intraradices*, *Funneliformis mosseae*, and *Funneliformis geosporum*	High temperature	Enhancement in plant attributes, photosynthetic transpiration rate, and pigments	Mathur et al. [18]
*Triticum aestivum*	*Rhizophagus irregularis*, *Funneliformis mosseae*, *Funneliformis geosporum*, and *Claroideoglomus claroideum*	High temperature	Enhancement in nutrient uptake and grain numbers	Cabral et al. [19]
*Zea mays*	*Glomus tortuosum*	Temperature stress	Increase in N, P, K, and Cu level in the shoot, and N, P, Ca, and Zn in the root and nitrate reductase (NR) activity and nutrient level	Liu et al. [20]
*Zea mays*	*Glomus tortuosum*	Cold stress	Higher amino acid concentrations	Zhu et al. [21]
*Elymus nutans* Griseb.	*Funneliformis mosseae*	Cold stress	Rise in antioxidant enzymes photosynthetic pigments and plant growth	Chu et al. [22]
*Hordeum vulgare* L.	*Glomus versiforme* and *Rhizophagus irregularis*	Cold stress	Antioxidants, osmoprotectants enhances, plant growth improvises, potassium uptake, membrane constancy, and phenolics metabolism, which raises the survival rate	Hajiboland et al. [23]
*Cucumis sativus* L	*Rhizophagus irregularis*	Cold stress	Enhancement in photosynthetic efficiency increase in their carbon sink	Ma et al. [24]
Grapevine (*Vitis vinifera* L.)	*Rhizoglomus irregulare* and *Funneliformis mosseae*	High-temperature stress	Improvement in growth rate and substrate carbon conversion efficiency, determined by calorespirometric readings and stomatal conductance	Nogales et al. [25]
*Zea mays* L.	*Funneliformis*	High temperature	Regulated photosystem (PS) II heterogeneity	Mathur and Jajoo [26]
*Solanum lycopersicum* *Capiscum annuum* *Cucumis sativus*	*Rhizophagus irregularis*	High-temperature stress	Increase in vigor, productivity, and fruit quality	Reva et al. [27]
*Saccharum arundinaceum*	*Glomus* spp.	Drought	Increased levels of antioxidant enzymes and compounds, phenolics, glutathione, chlorophyll, and plant biomass in the leaves	Mirshad and Puthur [28]
*Triticum aestivum*	*Glomus mosseae*	Drought	Chlorophyll concentration, antioxidant enzymes, ascorbic acid, N, P, and K content increase	Rani [29]
*Ipomoea batatas*	*Glomus* spp.	Drought	Osmoprotectants adjust osmotic potential	Yooyongwech et al. [30]
*Lycopersicon esculatum* *Capsicum annuum*	*Rhizophagus irregularis* and *Rhizophagus fasciculatus*	Drought	Increase in biomass, root and shoot length, photosynthetic pigment, and lower proline concentration	Padmavathi et al. [31]
*Solanum lycopersicum*	*Funneliformis mosseae* and *Rhizophagus irregularis*	Drought	Increase in plant height, stomatal conductance, water use efficiency index, biomass, proline level, decreased ROS, and ABA level in leaf and root enhancements	Chitarra et al. [32]
*Triticum aestivum* L.	*Glomus mosseae*, *Glomus fasciculatum*, and *Gigaspora decipiens*	Drought	Enhancement in plant growth parameters and photosynthetic pigments	Pal and Pandey [33]
*Digitaria eriantha*	*Rhizophagus irregularis*	Drought	Enhancement in shoot dry weight, stomatal conductance, lipid peroxidation, and ROS in shoot and root	Pedranzani et al. [34]
*Triticum durum*	*Rhizophagus intraradices*	Drought	Increase in grain biomass, micronutrients, and gliadins in grains	Goicoechea and Antol and Goicoechea et al. [35, 36]
*Poncirus trifoliate*	*Funneliformis mosseae* and *Paraglomus occultum*	Drought	Increased in root weight and length, higher fructose and glucose level but lower sucrose level, sucrose phosphate synthase (SPS) activity. Proline accumulation in roots	Zhang et al. [37]
*Cupressus arizonica*	*Rhizophagus irregularis* and *Funneliformis mosseae*	Drought	Improved growth and water deficit-induced hydrogen peroxide and malondialdehyde	Zhang et al. [38]
*Ephedra foliata Boiss*	*Glomus etunicatum*, *Rhizophagus intraradices*, and *Funneliformis mosseae*	Drought	Increased antioxidant enzyme activity, proline, glucose, total soluble protein, and plant hormone levels, as well as improved nitrogen metabolism	Wu et al. [39]
*Zea mays* L.	*Rhizophagus irregularis*	Drought	AM plant roots had diamine oxidase, which converted put into aminobutyric acid (GABA)	Aalipour et al. [40]
*Ceratonia siliqua*	*Glomus*, *Gigaspora*, *Acaulospora*, and *Entrophospora*	Drought	Increase in plant growth, nutrient level, stomatal conductance PSII system, water content, and organic solutes and decrease in ROS and lipid peroxidation	Hu et al. [41]
*Catalpa bungee* C.A.Mey	*Rhizophagus intraradices*	Drought	Enhancement in water content, biomass, photosynthetic pigment plant hormones except ABA, and zeatin in leaves and decrease in reactive oxygen species (ROS) in leaves. Improved root morphology and increasing the glomalin-related soil protein (GRSP) contents	Chen et al. [42]
*Cinnamomum migao*	*Glomus lamellosum* and *Glomus etunicatum*	Drought	Increase in antioxidant enzymes and osmoprotectants and reduction in malondialdehyde (MDA) level in the seedlings	Chen et al. [43]
*Sesamum indicum* L.	*Funneliformis mosseae* and *Rhizophagus intraradices*	Drought	Enhancement of oil and seed yield, total soluble protein, phosphorus level in leaf, photosynthetic pigments, and unsaturated fatty acids and decrease in level of saturated fatty acids	Xiaofeng et al. [44]
*Lonicera japonica* Thunb.	*Rhizophagus intraradices* and *Glomus versiforme*	Cd	Lower levels of Cd in shoots and roots; roots have higher Cd concentrations than shoots but lower Cd concentrations than shoots	Gholinezhad and Darvishzadeh [45]
*Solanum lycopersicum* L.	*Funneliformis mosseae*, *Rhizophagus intraradices*, and *Claroideoglomus etunicatum*	Cd	Malonaldehyde and ROS levels are decreased; the immune system is strengthened, and Cd stress is well protected	Jiang et al. [46]
*Cajanus cajan* L.	*Rhizophagus irregularis*	Metals—cadmium and zinc	Root biomass, macro- and micronutrients, and proline formation all increased	Hashem et al. [47]
*Zea mays* L.	*Glomus intraradices*	Heavy metal: cadmium	Combined effects on soil alkalinization, Cd immobilisation, and Cd phytoavailability	Garg and Singh [48]
*Trigonella foenumgraecum*	*Glomus monosporum*, *Glomus clarum*, *Gigaspora nigra*, and *Acaulospora laevis*	Metals—cadmium	Enhacement in antioxidant enzyme activities and malondialdehyde content	Liu et al. [49]
*Trigonella foenumgraecum*	*Glomus monosporum*, *Glomus clarum,* and *Gigaspora nigra*	Cd	Phytostabilization	Abdelhameed and Rabab [50]
*Glycine max*	*Rhizophagus irregularis*	Cd	In both HX3 and HN89 plants, there are no impacts on the accumulation or translocation of Cd	Abdelhameed and Metwally [51]
*Helianthus annuus*	*Glomus mosseae* and *Glomus intraradices*	Cr, Mn, Ni, Cu, Zn, Al, Pb, Co, Mo, Fe, and Si	Maximum amounts of glomalin and metal uptakes in the plant	Cui et al. [52]
*Zea mays*	*Rhizophagus fasciculatus*, *Rhizophagus intraradices*, *Funneliformis mosseae*, and *Glomus aggregatum*	Cd, Cr, Ni, and Pb	Phytoextraction	Sayın et al. [53]
*Phragmites australis*	*Funneliformis mosseae*	TiO_2_NPs	Ti is being more concentrated in the roots. Enhanced the accumulation of Ti in roots and altered the distribution of Ti in reeds	Singh et al. [54]
*Cynodon dactylon*	*Funneliformis mosseae* and *Diversisporas purcum*	Pb, Zn, and Cd	Modifications to the plant's HM content and deposition traits	Xu et al. [55]
*Medicago sativa*	*Glomus aggregatum*, *Glomus intraradices*, *Glomus elunicatum*, and *Glomus versiforme*	Cd	Alfalfa cultivated on Cd-polluted soil had less cadmium uptake	Zhan et al. [56]
*Medicago truncatula*	*Rhizophagus irregularis*	Pb	The amount of water-soluble Pb compound was reduced. AM injection reduced the amount of water-soluble Pb compound	Zhang et al. [57]
*Phragmites australis*	*Rhizophagus irregularis*	Cu	Phytorhizoremediation	Wu et al. [58]
*Sorghum vulgare*	*Acaulospora fragilissima*, *Acaulospora saccata*, *Claroideoglomus etunicatum*, *Pervetustus simplex*, *Rhizophagus neocaledonicus*, *Scutellospora ovalis*, and *Rhizophagus neocaledonicus*	Heavy metal: ultramafic soils (Fe, Mn, Ni, Cr, and Co)	Increase in root colonization, root and shoot dried weight, and P content	Crossay et al. [59]
*Solanum lycopersicum* L.	*Funneliformis mosseae*	*Cladosporium fulvum* Cooke 1883	Total chlorophyll content and net photosynthesis rate rise with increasing fresh and dry weight	Wang et al. [60]
*Saccharum offcinarum* L.	*Gigaspora margarita*	*G. etunicatum* and *Scutellospora fulgida*	Increased plant biomass, plant growth, and plant physiological characteristics	Manjunatha et al. [61]
*Astragalus adsurgens* var. *Shanxi Yulin*	*Claroideoglomus etunicatum*, *Glomus versiforme*, and *Funneliformis mosseae*	*Erysiphe pisi* DC 1805	Boosted the shoot and root growth of standing milkvetch despite increasing susceptibility when they were present in the roots towards powdery mildew	Liu et al. [62]
*Lycopersicon esculentum*	*Glomus* spp.	*Fusarium oxysporum f. sp. lycopersici*	Synthesis of antimicrobial compounds increased plant dry weight, growth, N, P, K, chlorophyll content, and yield	Kumari and Prabina [63]
*Capsicum annum*	*Glomus* spp.	*Pythium aphanidermatum*	Decreased infection and enhanced crop plant growth and yield	Kumari and Srimeena [64]
*Glycine max* (L.) Merr	*Rhizophagus irregularis*	*Macrophomina phaseolina*	Heighten the plant and increase the quantity of functional leaves	Spagnoletti et al. [65]

## Data Availability

The data will be available upon request.
